# Genetically Encoded Sensor Cells for the Screening of Glucocorticoid Receptor (GR) Effectors in Herbal Extracts

**DOI:** 10.3390/bios11090341

**Published:** 2021-09-16

**Authors:** Chungwon Kang, Soyoun Kim, Euiyeon Lee, Jeahee Ryu, Minhyeong Lee, Youngeun Kwon

**Affiliations:** 1 Department of Biomedical Engineering, Dongguk University, Seoul 04620, Korea; iu8974@dgu.ac.kr (C.K.); youn3256@gmail.com (S.K.); euiyeon.lee@dongguk.edu (E.L.); annlove7@dongguk.edu (J.R.); 2017126656@dgu.ac.kr (M.L.); 2 Department of Chemistry and Chemical Biology, Rutgers University, Piscataway, NJ 08854, USA

**Keywords:** cortisol, glucocorticoid receptor (GR), cell-based sensor, drug screening, signal peptide reconstitution, conditional protein splicing (CPS)

## Abstract

Although in vitro sensors provide facile low-cost ways to screen for biologically active targets, their results may not accurately represent the molecular interactions in biological systems. Cell-based sensors have emerged as promising platforms to screen targets in biologically relevant environments. However, there are few examples where cell-based sensors have been practically applied for drug screening. Here, we used engineered cortisol-detecting sensor cells to screen for natural mimetics of cortisol. The sensor cells were designed to report the presence of a target through signal peptide activation and subsequent fluorescence signal translocation. The developed sensor cells were able to detect known biological targets from human-derived analytes as well as natural product extracts, such as deer antlers and ginseng. The multi-use capability and versatility to screen in different cellular environments were also demonstrated. The sensor cells were used to identify novel GR effectors from medicinal plant extracts. Our results suggest that decursin from dongquai had the GR effector function as a selective GR agonist (SEGRA), making it a potent drug candidate with anti-inflammatory activity. We demonstrated the superiority of cell-based sensing technology over in vitro screening, proving its potential for practical drug screening applications that leads to the function-based discovery of target molecules.

## 1. Introduction

There is always a strong need for improved analytical strategies that can offer new avenues to screen for molecules of interest [1,2]. Current approaches to screen drug candidates are often based on in vitro competitive binding assays and immunoassays [3,4,5,6]. Although these in vitro assays offer facile low-cost ways of screening, they have some limitations. First, in vitro screenings are often performed using purified analytes, whose interactions may not accurately represent the interactions in a biological context, where a myriad of biologically active molecules are present. Second, antibody-based immuno-screenings examine the analytes based on their structures rather than their biological functions, seriously restricting the pool of analytes that can be screened to only molecules with structural similarities. Therefore, screening analytes based on their biological functions in their native working environment is essential to effectively identify drug candidates.

Cell-based sensing approaches can overcome the aforementioned limitations while avoiding ethical issues related to animal testing [7,8], thereby representing an effective alternative to in vitro assays. Cell-based sensors are often developed as genetically encoded biosensors using native receptors or enzymes as the molecular recognition components and fluorescent or luminescent proteins as the optical reporters. Current optical reporting systems are based either on fluorescence or bioluminescence resonance energy transfer (FRET/BRET) or on bi-molecular fluorescence or luminescence complementation (BiFC/BiLC) [9,10,11]. Although FRET-based reporters allow for the direct visualization of protein–protein interactions inside living cells, they often yield false-positive/negative results because of spectral bleed-through or low energy transfer efficiency [12,13]. BiFC is based on fluorescence activation caused by the reassembly of two complementary non-fluorescent fragments of a fluorescent protein (FP) that are brought in close proximity to each other through an interaction between proteins fused to the fragments [9]. The two complementary fragments are not fluorescent on their own when kept apart; therefore, a high contrast can be obtained. Despite its popularity, BiFC has certain disadvantages [14,15,16]. The split fragments have considerable affinity to each other and tend to reassemble autonomously, yielding a false-positive signal. Moreover, they need to be folded into the correct tertiary structure to be activated, which is considerably time consuming and often results in misfolded proteins inducing false-negative signals. Therefore, designing split fragments that can refold into a functional reporter requires considerable amounts of time and effort.

In order to overcome the limitations of BiFC and FRET-based approaches, we have previously designed a reporting system based on an intein-mediated reconstitution of the signal peptide [17,18,19]. Complemented split-inteins self-catalytically react to form and break specific peptide bonds without requiring an energy source or cofactors [20,21]. The reconstituted signal peptide upon activation translocates the fluorescent cargo to indicate the presence of the target. Split-signal peptides are instantly activated through covalent bond formation without a tedious refolding step and cannot be activated by merely binding the split-fragments; therefore, this approach can address the limitations of BiFC and FRET-based reporting systems. These reporter systems have been designed into cell-based sensors that can detect various bioactive targets, including Ca^2+^, cortisol, and rapamycin [17,18,19]. These sensor cells demonstrated excellent performance in screening targets with enhanced sensitivity with a short response time. The sensor cells also exhibited abilities to detect functional analogues of targets while discriminating their structural analogues without functional similarities. 

Here, we built and characterized cell-based sensors to demonstrate their potential in practical drug screening applications. Fast-responding cortisol-detecting sensor cells that can screen for cortisol-like GR effectors were selected for the study. Cortisol, known as a steroidal stress hormone, is an endogenous glucocorticoid (GC) and a ligand of the glucocorticoid receptor (GR). Cortisol and its synthetic analogues are marketed as strong anti-inflammatory agents; however, they have adverse side-effects, such as skin atrophy and muscle weakness [22,23]. Therefore, it is important to identify novel GR effectors with anti-inflammatory activity and minimal side-effects. Although there are several very sensitive electrochemical immune-sensors developed for cortisol detection, they are not suitable for this purpose because they can only detect cortisol based on its structure [24,25,26]. Cell-based sensors provide an effective alternative to the current screening platforms in identifying natural mimetics of cortisol from natural sources. 

In this study, we evaluated cell-based sensor cells focusing on their multi-use capabilities as well as target identification from complex biological samples. We developed sensors from two different cell lines, enabling target screening in diverse cellular environments. Furthermore, we screened medicinal herb extracts with unidentified biological effects to identify GR effectors. Sensor cells responded to dongquai extracts and decursin was identified as a GR effector. Further analysis revealed that decursin functions as a selective GR agonist (SEGRA) with large therapeutic potential (Scheme S1). This study demonstrated the use of engineered sensor cells for the screening of unknown naturally derived compounds based on their functions to identify molecules with desired functionality.

## 2. Materials and Methods

### 2.1. General Procedure

General chemicals of the best available grade were obtained from Sigma-Aldrich (St. Louis, MO, USA). Fetal bovine serum (FBS) was purchased from Thermo Fisher Scientific (Waltham, MA, USA) and Dulbecco’s modified Eagle’s medium (DMEM) was procured from Welgene (Daegu, Korea). Triamcinolone acetonide and cyproterone acetate (CPA) were obtained from TCI chemical (Tokyo, Japan). Ginseng and deer antler extracts were purchased from Dongguk University Medical Center (Goyang, Korea). Confocal fluorescence images were captured using an Eclipse Ti confocal from Nikon Instruments (Tokyo, Japan) with excitation wavelengths of 358, 488, and 594 nm and corresponding emission filters. The fluorescence intensity was analyzed using the Nikon NIS-Element BR 4.60 software from Nikon Instruments (Tokyo, Japan) and the Image J software from the U.S. National Institutes of Health (Bethesda, MD, USA).

### 2.2. Preparation of Herbal and Plant Extracts

Dried herbs and plants were purchase from a local market, Gyeongdong market, and double boiled in 70% ethanol to extract their components. The ratio of dried herbs or plants to solvent (*w*/*v*), boiling temperature, and the extraction time were different for each sample, as described in Table S1. Each extract was successively filtered using a Whatman grade no. 1 filter paper and a 0.45 µm pore filter. The filtered extracts were lyophilized, and the lyophilized powders were dissolved in water to prepare stock solutions with the same concentration of 1 mg/mL. The final concentration used to treat the sensor cells was 1 μg/mL.

### 2.3. Sample Collection and Preparation to Detect Salivary Cortisol

For the detection of salivary cortisol, saliva was collected from three healthy individuals 10 min after waking up in the morning. The preservative-free saliva was immediately placed at −20 °C. Collected saliva samples were centrifuged and filtered using a 0.22 µm filter and preserved at −20 °C.

### 2.4. Screening of GR Effectors Using Sensor Cells

HeLa cells were grown in a 35 mm confocal dish and transiently transfected with the pRJH013 plasmid. Sensor probes were constitutively expressed at 37 °C for 24 h and the sensor cells were treated with the analytes. The cells were washed with PBS and their nuclei were stained with Hoechst 33,342 (8 µM) before or after treatment with analytes, as needed. The fluorescence images of sensor cells were captured and then analyzed using the Image J software to determine the fluorescence signal translocation. At least 3 sets of experiments were performed and over 20 cells were analyzed in each experiment.

### 2.5. Western Blotting

For the Western blot analysis, sensor cells were prepared in 100 mm dishes and stimulated with analytes for 2 h. Sensor cells were then lysed and analyzed using SDS-PAGE. The formation of the spliced product was assessed by Western blotting using an anti-Flag antibody.

### 2.6. Competitive In Vitro GR Binding Assay

In vitro GR binding affinity was measured using the PolarScreen GR competitor assay red kit from Thermo Fisher scientific (Waltham, MA, USA). The assay was performed according to the manufacturer’s instructions. Fluorescence polarization was measured using a Tecan Spark microplate reader (Männedorf, Switzerland).

### 2.7. Statistics

All data are presented as mean ± SEM. Data were analyzed and figures were drawn using GraphPad Prism 8.0 (San Diego, CA, USA). *p*-value < 0.05 was considered significant.

### 2.8. Reporter Gene Assay

HeLa cells were grown in a 96-well white plate in DMEM containing 5% FBS without phenol red for 24 h and individually transfected with either pGL4.36 (luc2P/MMTV/hygro) or pGL4.32 (luc2P/NF-κB-RE/hygro) from Promega (Madison, WI, USA). Cells were individually stimulated with each analyte. The luciferase activity was developed using the ONE-Glo luciferase assay system from Promega (Madison, WI, USA) and detected using the Cytation 5 from BioTek instruments (Winooski, VT, USA). The assays were performed following the manufacturer’s protocol.

## 3. Results

### 3.1. Fabrication and Characterization of Genetically Encoded Sensor Cells

**Figure 1:**
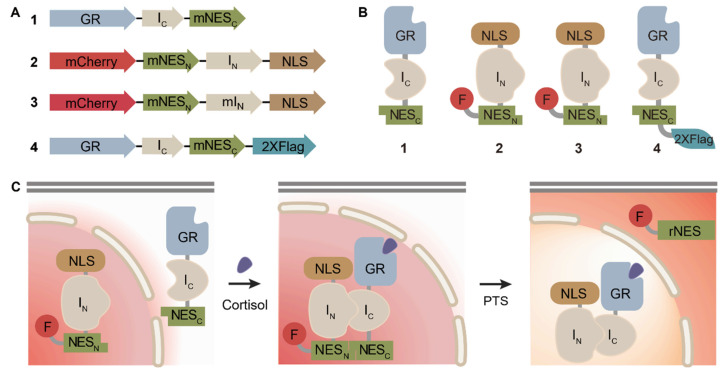
Design and working mechanisms of the cortisol-detecting sensor cells. The domain architecture (**A**) and schematic illustration (**B**) of fusion proteins used in this study; GR, glucocorticoid receptor; I_N_, N-intein; I_C_, C-intein; NLS, nuclear localization signal; NES, nuclear export signal. (**C**) Two fusion proteins containing each split-intein are located in two different cellular compartments. The target, cortisol, stimulates the nuclear translocation of GR to trigger conditional protein splicing (CPS), which results in reconstitution of the NES peptide. The reconstituted signal peptide delivers the fluorescent protein to the cytosol, thereby reporting the existence of the target molecule.

In order to screen natural products for GR effectors, we prepared a sensing platform that can report the interaction between the analytes and GR in living cells through fluorescence translocation. Previously reported sensing probes constructed with GR as a target recognition element and inactivated split-signal peptides conjugated to fluorescent cargo as a reporter element were used in this study [11]. The cortisol-detecting probes were prepared using the fast-reacting Nostoc punctiforme (Npu) DnaE split-inteins and the split nuclear export signal (NES) peptide (Figure 1A,B). Two fusion proteins were prepared; each contains the N- or C-terminal fragment of split-intein along with the corresponding N- or C-terminal fragment of split NES peptide as the extein. GR was inserted at the N-terminus of C-intein (I_C_) containing fusion protein to generate the gene encoding protein **1**, GR-I_C_-NES_C_. The mCherry and nuclear localization signal (NLS) genes were inserted at N- and C-terminus of N-intein (I_N_) containing fusion protein, respectively, to generate the gene encoding protein **2**, mCherry-NES_N_-I_N_-NLS. A non-functional mutant fusion protein **3**, mCherry-NES_N_-mI_N_-NLS, was prepared by replacing the first amino-acid of N-intein from Cys to Ala, thereby abolishing the protein splicing activity. Flag-tagged fusion protein **4**, GR-I_C_-NES_C_-Flag, was also prepared for Western blot analysis. Genetically encoded sensor cells were prepared by transiently transfecting HeLa cells with a pBI-CMV1 vector containing two constitutive promoters encoded with two fusion proteins, **1** and **2**. Protein **2** containing the fluorescence signal is initially located in the nucleus of the sensor cells owing to the NLS peptide (KRPAATKKAGQAKKKKLD), whereas protein **1** is located in the cytosol. Cortisol binds to GR and prompts the nuclear-translocation of protein **1** to trigger an intein-mediated conditional protein splicing (CPS) reaction, resulting in the reconstitution of the NES signal peptide (KVYPIILRLCFNLSL) [19,27]. The reconstituted signal peptide is instantly activated without tedious refolding steps with the consequent translocation of the fluorescent cargo to the cytosol, reporting the presence of the target, cortisol (Figure 1C).

**Figure 2:**
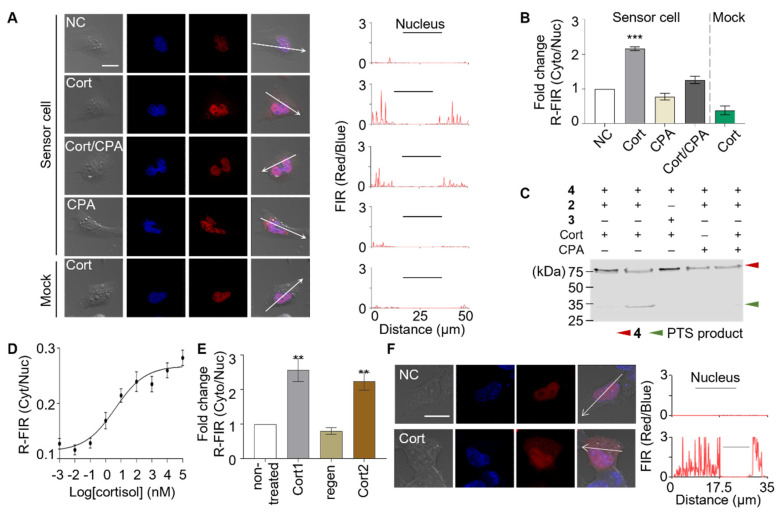
Characterization of cortisol (Cort)-detecting sensor cells. (**A**) Sensor cells co-expressing fusion proteins **1** and **2** exhibited red fluorescence from mCherry in the nucleus prior to stimulation (row 1). The fluorescence signal was translocated to the cytosol upon Cort treatment (row 2). Mock sensors containing fusion proteins **1** and **3** did not respond to Cort (row 3). The passive GR antagonist, CPA, inhibited the Cort-induced signal translocation (row 4 and 5) (scale bar = 20 µm). (**B**) Fold-change in the ratio of red fluorescence intensities of the cytoplasm to that of the nucleus (R-FIR Cyto/Nuc) over untreated control sensor cells is calculated. Data were analyzed by one-way ANOVA using the Tukey multiple comparison test (* *p* < 0.05, ** *p* < 0.01, *** *p* < 0.001). (**C**) Western blot analysis revealed the formation of reconstituted signal peptides via protein trans-splicing. (**D**) Dose–response of R-FIR Cyto/Nuc signal was plotted and the limit of detection (LOD) was determined to be 1.6 nM. (**E**) The Cort-treated sensor cells were spontaneously regenerated and used multiple times to detect Cort without a notable loss of function. (**F**) HEK293T cell-derived sensor cells were treated with Cort and exhibited fluorescence signal translocation.

Prior to the screening of unknown analytes, we assessed the target detection capability of the sensor cells and their functioning mechanisms. HeLa cells were genetically modified to express two fusion proteins, **1** and **2**, thereby generating the sensor cells. Non-functional mock sensor cells were prepared by genetically modifying HeLa cells to express fusion proteins **1** and **3**. Sensor cells and mock sensor cells were both treated with cortisol and observed using fluorescence microscopy. Red fluorescence signals were initially located in the nucleus and then translocated to the cytosol in the sensor cells upon treatment with cortisol (Figure 2A, rows 1 and 2). The graph shown on the right-hand side of the images was obtained by evaluating the red-to-blue fluorescence intensity ratio (FIR red/blue) across the sensor cell. The mock sensor cells did not respond to the cortisol treatment, demonstrating that the presence of the C1 amino acid sequence in N-intein was crucial for the functioning of the sensor cells. This indicated that the red-fluorescence translocation in sensor cells is induced by the formation of covalent bonds through the protein trans-splicing (PTS) reaction (Figure 2A, row 3). The response of the sensor cells was quantitatively analyzed by determining the ratio of the red fluorescent intensity of the cytoplasm and to that of the nucleus (R-FIR Cyto/Nuc) (Figure 2B). The specificity of the GR effector interaction was assessed by co-treating the sensor cells with a passive GR antagonist, cyproterone acetate (CPA). Although the passive GR antagonist binds to GR, it does not induce nuclear translocation (Scheme S1). As expected, the CPA treatment alone did not have an apparent effect on the sensor cells; however, it inhibited the cortisol-mediated fluorescence translocation upon co-treatment (Figure 2A row 5, Figure 2B). This result elucidated that the observed fluorescence signal translocation was induced by the specific interaction between the GR and cortisol. Western blotting analysis confirmed the formation of PTS reaction products, indicating the formation of covalent bonds that conjugated and activated the signal peptide (Figure 2C).

### 3.2. Evaluating the Performance of the Sensor Cells

The performance of the sensor cells was evaluated to demonstrate their potential in practical drug screening applications. First, their sensitivity was determined by varying the target concentrations and monitoring the sensor cell responses (Figure 2D and Figure S1). Sensor cells exhibited excellent sensitivity with a limit of detection (LOD) of 1.6 nM; the LOD is the concentration at which the signal ratio is three times the standard deviation of the negative control mean values. Furthermore, the sensor cells showed excellent reproducibility with a CV (coefficient of variation) of 7%. This result demonstrates that these sensor cells exhibited sufficient sensitivity to facilitate the screening of cortisol and GR effectors in low-content analytes, including human-derived samples. Then, we tested the sensor cells by monitoring their detection of the cortisol in human saliva. Upon being challenged with filter-sterilized saliva samples, the sensor cells exhibited fluorescence translocation with an approximate 2-fold increase in R-FIR Cyto/Nuc (Figure S2), which is equivalent to a cortisol concentration of 13 nM. This result is consistent with previously reported concentrations of salivary cortisol [26,28]. The response speed was also monitored; the sensor cells rapidly responded to the target with a minimal response time of less than 5 min and showed maximum signal in 1 h (Figure S3). These results demonstrate the excellent performance of sensitive and fast-responding sensor cells that can screen for targets in biological fluids.

Another desirable characteristic for biosensor implementation is reusability with low-effort sensor regeneration or renewal [29,30]. We investigated whether these sensor cells are able to repetitively report the presence of the target. After being exposed to cortisol, the sensor cells were autonomously regenerated in culture media by losing fluorescence signals in cytosol. Subsequently, the regenerated sensor cells were rechallenged with the target. The following R-FIR Cyto/Nuc values were determined at each step: 0.10 before the treatment, 0.25 after the first cortisol treatment, 0.08 after renewal, and 0.22 after the second cortisol treatment (Figure 2E and Figure S4). This indicated that the sensor cells are capable of repetitive detection with simple autonomous regeneration steps. The regeneration mechanism is probably based on the degradation and regeneration of intracellular proteins, including the sensing and reporting units. 

The functions of the sensor cells could differ based on cell type. In order to test sensor cell development in various cell types, we introduced the sensing system into human embryonic kidney cells (HEK293T) and evaluated their cortisol-detecting capabilities. The HEK293T-derived sensor cells responded to cortisol with the R-FIR Cyto/Nuc values of 0.04 and 0.22, before and after treatment, respectively (Figure 2F and Figure S5). It was notable that the level of background signal was minimized in the HEK293T-derived sensor cells. This result suggests that the sensor cells can be generated using multiple cell types, thereby enabling the screening of targets in varying cellular environments.

**Figure 3:**
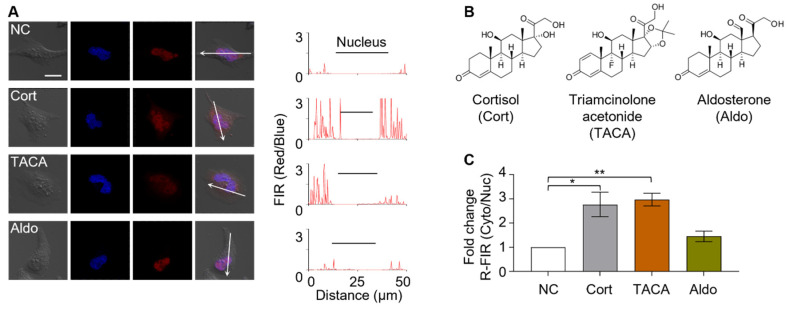
Evaluation of Cort-detecting sensor cells for function-based screening. (**A**) The sensor cells responded to Cort and the GR agonist, triamcinolone acetonide (TACA), while discriminating against the structural analogues, aldosterone (Aldo) (scale bar = 20 µm). (**B**) The structures of Cort, TACA, and Aldo. (**C**) Fold change in the ratio of red fluorescence intensity from the cytoplasm to that of the nucleus (R-FIR Cyto/Nuc) over untreated control sensor cells is calculated. Data were analyzed by one-way ANOVA using the Tukey multiple comparison test (* *p* < 0.05, ** *p* < 0.01).

The sensor cells were then tested for target selectivity using two steroid compounds, triamcinolone acetonide and aldosterone (Figure 3B). Triamcinolone acetonide is a well-known synthetic agonist of GR. Aldosterone, a mineralocorticoid, is a structural analogue of cortisol that does not have GR-binding affinity. Sensor cells were individually treated with either cortisol, triamcinolone acetonide, or aldosterone (Figure 3A,C). Sensor cells responded to cortisol and triamcinolone acetonide, exhibiting comparable fluorescence translocation with R-FIR Cyto/Nuc values of 0.29 for both targets. In contrast, the sensor cells showed a marginal response to aldosterone, a non-functional structural analogue of cortisol, yielding an R-FIR Cyto/Nuc value of 0.17, indicating that the generated sensor cells can distinguish between GR effectors and non-functional structural analogues. Therefore, these sensor cells provide a useful platform to screen for GR effectors in a complex mixture containing several steroidal compounds.

### 3.3. Screening Herbal Medicines to Identify GR Effectors

Phytochemicals in medicinal plants are known to possess multiple therapeutic effects, such as anti-bacterial, anti-tumor, blood-sugar-lowering, and anti-inflammatory properties [31]. Owing to their potency, medicinal plants are often used to boost strength and endurance in oriental medicine [32,33]; there is also growing interest in the potential health benefits of dietary phytochemicals [34]. However, biological roles of most phytochemicals in the human body have not been elucidated. Medicinal plants and herbal extracts have been considered to be a rich source of natural bioactive compounds. Many herbal extracts have been reported to improve the immune system, decrease inflammation, and relieve the effects of stress; therefore, it is a logical approach to search medicinal plant extracts to identify natural mimetics of cortisol [33].

**Figure 4:**
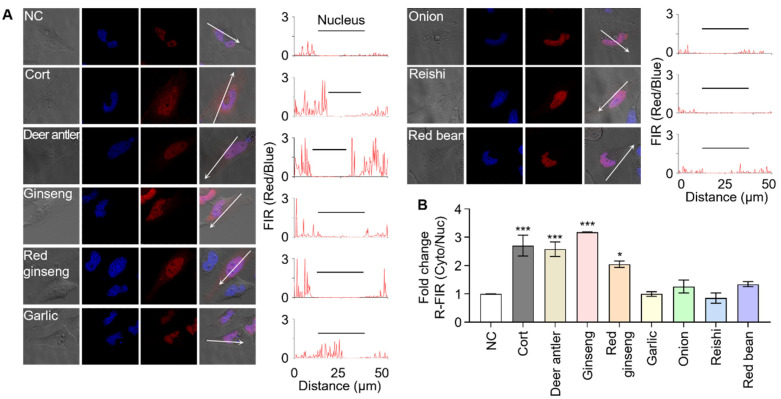
Screening natural product extracts containing cortisol or saponin family compounds. (**A**) The sensor cells were individually challenged with seven different extracts of cortisol or saponin-containing natural products. The sensor cells responded to the cortisol-containing deer antler extract as well as the Rg1- and Re-containing ginseng and red ginseng extracts (scale bar = 20 µm). (**B**) Fold change in the ratio of red fluorescence intensity from the cytoplasm to that of the nucleus (R-FIR Cyto/Nuc) over untreated control sensor cells is calculated. Data were analyzed by one-way ANOVA using the Tukey multiple comparison test (* *p* < 0.05, ** *p* < 0.01, *** *p* < 0.001).

First, deer antlers and ginseng extracts were screened; deer antlers are known to possess native cortisol and ginseng possesses multiple GR effectors, such as ginsenosides Rg1 and Re [35,36,37,38]. Sensor cells treated with either deer antlers or ginseng extracts reported the presence of GR effectors through fluorescence translocation with R-FIR Cyto/Nuc values of 0.26 or 0.32, respectively (Figure 4). Our results indicate that the sensor cells are suitable to screen natural product extracts for GR effectors; therefore, we expanded our screening to include more analytes. We analyzed red ginseng extracts and saponin-rich plant extracts because the GR effectors in ginseng, Rg1 and Re, belong to the saponin family. Extracts of onion, garlic, red bean, and reishi mushroom were chosen as the saponin-containing plant extracts. The results reveal that only the red ginseng extract induced signal translocation in sensor cells, suggesting that other saponin family compounds in the tested extracts might not be potent GR effectors or might not to be present in working concentrations [39,40].

**Figure 5:**
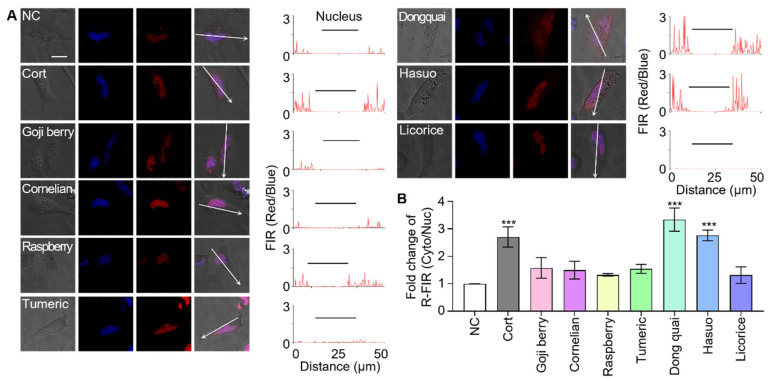
Screening medicinal plant extracts using sensor cells. (**A**) The sensor cells were individually challenged with seven different extracts of medicinal plants. The sensor cells responded to dongquai and hasuo extracts (scale bar = 20 µm). (**B**) Fold change in the ratio of red fluorescence intensity from the cytoplasm to that of the nucleus (R-FIR Cyto/Nuc) over untreated control sensor cells is calculated. Data were analyzed by one-way ANOVA using the Tukey multiple comparison test (* *p* < 0.05, ** *p* < 0.01, *** *p* < 0.001).

Next, the following seven herbal extracts from frequently prescribed oriental medicines were selected as analytes: goji berry, cornelian berry, black raspberry, turmeric, dongquai, hasuo, and licorice (Table S1). Sensor cells were individually treated with the same amount of each plant extract and the fluorescence translocation was monitored. Sensor cells treated with dongquai and hasuo both reported the presence of GR effectors in their analytes by fluorescence translocation with R-FIR Cyto/Nuc values of 0.32 and 0.28, respectively (Figure 5). Sensor cells treated with goji berry, cornelian berry, black raspberry, turmeric, and licorice exhibited negligible signals, with R-FIR Cyto/Nuc values of 0.16, 0.15, 0.13, 0.15, and 0.13, respectively. Notably, to our best knowledge, GR effectors have not been identified in dongquai and hasuo extracts before this study. Although licorice is known to possess 18β-glycyrrhetinic acid, which is an effector for GR and a mineralocorticoid receptor [41,42], no fluorescence translocation was observed in the sensor cells. This could be caused by low concentrations of active ingredients or ineffective extraction.

### 3.4. Identification of Active Components

**Figure 6:**
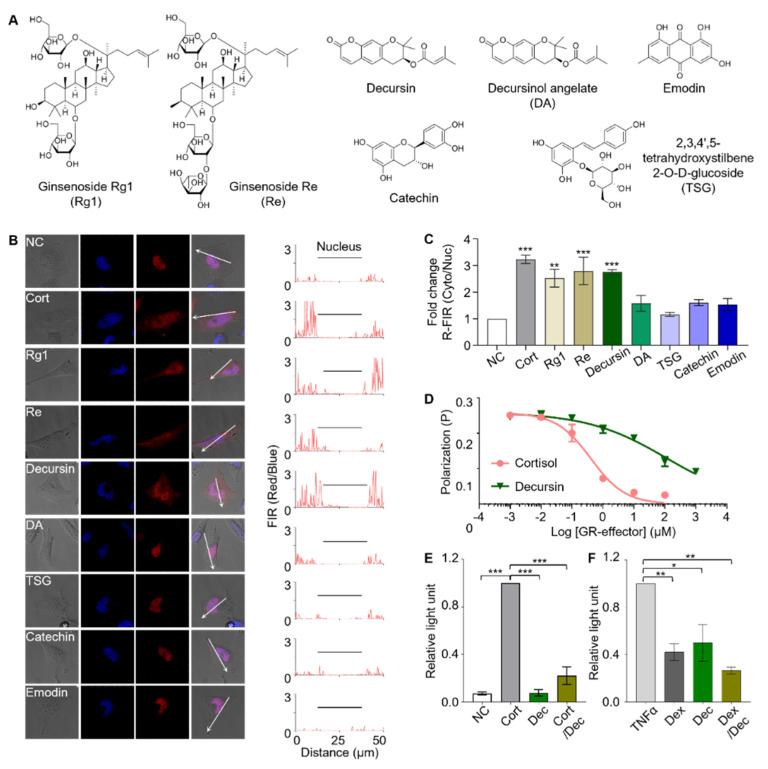
Screening the major components of ginseng, dongquai, and hasuo using sensor cells to identify GR effectors. (**A**) Structures of the major components in ginseng, dongquai, and hasuo. Rg1 and Re are components of ginseng, decursin and decursinol angelate (DA) are components of dongquai, 2,3,4′,5-tetrahydroxystilbene 2-O-D-glucoside (TSG), catechin, and emodin are components of Hasuo. (**B**) The sensor cells were challenged with seven major components derived from medicinal plants; they responded to Rg1, Re, and decursin (scale bar = 20 μm). (**C**) Fold change in the ratio of red fluorescence intensity from the cytoplasm to that of the nucleus (R-FIR Cyto/Nuc) over untreated control sensor cells is calculated. Data were analyzed by one-way ANOVA using the Tukey multiple comparison test (* *p* < 0.05, ** *p* < 0.01). (**D**) In vitro competitive binding assay demonstrating that decursin binds to GR with much lower affinity than that of cortisol. (**E**) Glucocorticoid response element (GRE)-mediated transactivation study revealed that decursin does not activate GRE-mediated transactivation. (**F**) The NF-κB-mediated trans-repression study revealed that decursin plays a role in NF-κB-mediated trans-repression (* *p* < 0.05, ** *p* < 0.01, *** *p* < 0.001).

In order to identify GR effectors present in the plant extracts, we analyzed the major components of the plant extracts using the sensor cells [43,44,45]. The following major components were identified from the extracts: Rg1 and Re from ginseng; decursin and decursinol angelate (DA) from dongquai; and 2,3,5,4′-Tetrahydroxystilbene-2-O-β-D-glucoside (TSG), catechin, and emodin from hasuo (Figure 6A). Sensor cells were individually treated with 1 µg/mL of each compound in an aqueous solution, and the fluorescence translocation was monitored (Figure 6B). Of the seven tested compounds, sensor cells responded to Rg1, Re, and decursin by reporting red fluorescence translocation from the nucleus to the cytosol with R-FIR Cyto/Nuc values of 0.25, 0.28, and 0.28, respectively (Figure 6C). Sensor cells showed marginal responses to DA, TSG, catechin, and emodin with R-FIR Cyto/Nuc values of 0.16, 0.12, 0.16, and 0.15, respectively. Our result confirm the previously reported function of Rg1 and Re as GR effectors. However, as for decursin, its bioactivity has been acknowledged to be exerted by androgen receptor- and estrogen receptor-mediated signaling [46,47,48]. Therefore, to our best knowledge, this work is the first to reveal the biological function of decursin as a GR effector.

The assays based on sensor cells showed that decursin is a GR effector that binds to GR and induces nuclear translocation. We further investigated the biological function of decursin to understand it in more detail. The binding of GR and decursin was verified in vitro using a competitive fluorescence polarization assay (Figure 6D). The result indicates that the in vitro binding of decursin to GR is much weaker than that of cortisol, whose K_D_ was three orders of magnitude greater. This weak in vitro binding affinity is probably the reason this effector has not been identified before.

The biological functions of GCs are exercised through the regulation of abundant GR-related gene cassettes. GR functions as a transcription factor through three modes of action depending on the effectors that binds to GR (Scheme S1). Depending on their roles in the regulation of gene expression, GR effectors can be categorized into GR-agonists, SEGRAs, or active antagonists. Therefore, further analysis was performed to identify the role of decursin in GR-mediated gene regulation using the following reporter gene assays: glucocorticoid-responsive element (GRE)-mediated transactivation; and NF-κB-mediated trans-repression. Decursin did not induce GRE-mediated transactivation and inhibited cortisol-induced GRE-mediated transactivation upon co-treatment (Figure 6E), indicating that decursin is probably not an agonist of GR. In contrast, the NF-κB-mediated tethered trans-repression assay revealed that decursin is an effective repressor of TNF-α induced NF-κB-mediated trans-repression (Figure 6F). The results of both assays interpreted together indicate that decursin is a SEGRA. The anti-inflammatory function of GR effectors is related to the repression of NF-κB-mediated trans-repression, whereas muscle atrophy is induced by the GRE-mediated transactivation [49,50]. Considering their therapeutic benefits, SEGRAs, which have NF-κB-mediated trans-repression activity without GRE-related activation, are potential drug candidates that can replace GR agonists.

## 4. Discussion

Cell-based sensors are a promising platform for various applications that require the screening of targets in their native environment. Sensor cells have often been utilized for the spatiotemporal analysis of molecular interactions and their dynamics [51,52]. Although the ability of sensor cells to screen for drug candidates has been reported, the examples are often limited to the screening of known and/or purified analytes. In this study, we prepared sensitive and fast-responding sensor cells that can screen targets based on their cellular functions while discriminating structural analogues without functional similarities. Sensor cells were developed using the fluorescence translocation reporting system involving the intein-mediated activation of signal peptide, which eliminates false-positive/negative signals originating from binding interactions or misfolded reporter proteins. Notably, the sensor cells required neither the use of any chemical reporters, such as chromogenic or luminogenic substrates, nor an external energy source, highlighting their suitability to screen complex chemicals without interference. Moreover, we also demonstrated the sustainability and multi-use capability of the sensor cells, revealing their autonomous regeneration without requiring extra treatment. Furthermore, the sensor cells were generated from multiple cell lines, demonstrating function-based screening in various cellular environments.

In addition to screening for targets and their functional analogues, the sensor cells allowed us to investigate the effects of inhibitors. The sensor cells demonstrated the ability to semi-quantitatively screen for cortisol in human-derived samples, revealing a concentration of 13 nM, similar to previously reported values. The sensor cells were also used to screen for GR effectors using medicinal plant or herb extracts that contain multiple phytochemicals with various biological activities. Our results newly identified dongquai and hasuo extracts to possess GR effector-activities. Further screening of their major components revealed a novel GR effector, decursin. Interestingly, although decursin exhibited very low binding affinity to GR in vitro, the sensor cells had a comparable response to decursin as that to cortisol. Further analysis elucidated that decursin functions as a SEGRA, which is known to possess anti-inflammatory activity without causing muscle atrophy. Decursin has been previously reported to be involved in androgen-receptor- and estrogen receptor-mediated signaling; therefore, further investigations are required to understand their integrated biological effect. This study allowed us to verify the potential of cell-based sensors for drug screening applications by demonstrating their unique characteristics compared to in vitro assays, leading to a more effective screening of target molecules.

## Data Availability

The data presented in this study are included in this published article and its additional files. All the data can be shared upon request by email.

## Supplementary Materials

The following are available online at https://www.mdpi.com/article/10.3390/bios11090341/s1, Scheme S1: A schematic illustration of the mode of GR gene regulation; Figure S1: Dose-dependent treatment of cortisol to sensor cells; Figure S2: Detection of salivary cortisol using sensor cell; Figure S3: Time-dependent treatment of cortisol to sensor cells; Figure S4: Renewability of sensor cell; Figure S5: Cortisol sensing using HEK293T derived sensor cell; Table S1: Table of medicinal plants and extraction conditions.
