# Supplementary material for: Genetically Encoded Sensor Cells for the Screening of Glucocorticoid Receptor (GR) Effectors in Herbal Extracts
Source: Biosensors (Basel). 2021 Sep 16;11(9):341. doi: 10.3390/bios11090341 (PMC8465347; doi:10.3390/bios11090341)
Supplement: Supplementary file 1 [file biosensors-11-00341-s001.zip › biosensors-1351301-supplementary.pdf]

biosensors

MDPI

Article

Genetically Encoded Sensor Cells for the Screening of Glucocorticoid Receptor (GR) Effectors in Herbal Extracts

Chungwon Kang [1], Soyoun Kim [1], Euiyeon Lee [1,2], Jeahee Ryu [1], Minhyeong Lee [1] and Youngeun Kwon [1,*]

[1] Department of Biomedical Engineering, Dongguk University, Seoul 04620, Korea; iu8974@dgu.ac.kr (C.K.); youn3256@gmail.com (S.K.); euiyeon.lee@dongguk.edu (E.L.); annlove7@dongguk.edu (J.R.); 2017126656@dgu.ac.kr (M.L.)
[2] Department of Chemistry and Chemical Biology, Rutgers University, Piscataway, NJ 08854, USA
* Correspondence: ykwon@dongguk.edu; Tel.: +82-31-961-5151

Protein sequence

GR-Npu$_C$-mNES$_C$ (1)
Expected Mass (kDa): 90.57
Sequence:
MDSKESLTPGREENPSSVLAQERGDVMDFYKTLRGGAT-
VKVSASSPSLAVASQSDSKQRRLLVDFPKGSVSNAQQPDLSKAVSLSMGLYMGETETK
VMGNDLGFPQQGQISLSSGETDLKLLEESIANLNRSTSVPEN-
PKSSASTAVSAAPTEKEFPKTHSDVSSEQQHLKGQTGTNGGNVKLYTTDQSTFDILQD
LEFSSGSPGKETNESPWRSDLLIDENCLLSPLAGEDDSFLLEGNSNEDCK-
PLILPDTKPKIK-
DNGDLVLSSPSNVTLPQVKTEKEDFIELCTPGVIKQEKLGTVYCQASFPGANIIGNKMS
AISVHGVSTSGGQMYHYDMNTASLSQQQDQKPIFNVIPPIPVGSENWN-
RCQGSGDDNLTSLGTLNFPGRTVFSNGYSSPSMRPDVSSPPSSSSTATTGPPPKLCLVCS
DEASGCHYGVLTCGSCKVFFKRAVEGQHNYLCAGRNDCIIDKIRRKNCPACRYRK-
CLQAGMN-
LEARKTKKKIKGIQQATTGVSQETSENPGNKTIVPATLPQLTPTLVSLLEVIEPEVLYAG
YDSSVPDSTWRIMTTLNMLGGRQVIAAVKWAKAIPG-
FRNLHLDDQMTLLQYSWMFLMAFAL-
GWRSYRQSSANLLCFAPDLIINEQRMTLPCMYDQCKHMLYVSSELHRLQVSYEEYLC
MKTLLLLSSVPKDGLKSQELFDEIRMTYIKELGKAIVKREGNSSQNWQRFYQLT-
KLLDSM-
HEVVENLLNYCFQTFLDKTMSIEFPEMLAEIITNQIPKYSNGNIKKLLFHQKLEIKIATR
KYLGKQNVYDIGVERDHNFALKNGFIASNCFNLSL

mCherry-mNES$_N$-Npu$_N$-NLS (2)
Expected Mass (kDa): 44.29
Sequence:
MVSKGEEDNMAIIKEFMRFKVHMEGSVNGHE-
FEIEGEGEGRPYEGTQTAKLKVTKGG-
PLPFAWDILSPQFMYGSKAYVKHPADIPDYLKLSFPEGFKWERVMNFEDGGVVTVTQ
DSSLQDGEFIYKVKLRGTNFPSDGPVMQKKTMGWEASSERMYPEDGALKGEIKQRL-
KLKDGGHYDAEVKTTYKAKKPVQLPGAYNVNIKLDITSHNEDYTIVEQHERAEGRHS
TGGMDELYKPRKVYPIILRLCLSYETEILTVEYGLLPIGKIVEKRIECTVYSVDNNGNI-
YTQPVAQWHDRGEQEVFEYCLEDGSLIRATKDHKFMTVDGQMLPIDEIFERELDLMR
VDNLPNIKIATRKYLGKQNVYDIGVERKRPAATKKAGQAKKKKLD

mCherry-mNES$_N$-mNpu$_N$-NLS (3)
Expected Mass (kDa): 44.25

Sequence:
MVSKGEEDNMAIIKEFMRFKVHMEGSVNGHE-FEIEGEGEGRPYEGTQTAKLKVTKGGPLPFAWDILSPQFMYG-SKAYVKHPADIPDYLKLSFPEGFKWERVMNFEDGGVVTVTQDSSLQDGEFI-YKVKLRGTNFPSDGPVMQKKTMGWEASSERMYPEDGALKGEIKQRL-KLKDGGHYDAEVKTTYKAKKPVQLPGAYNVNI-KLDITSHNEDYTIVEQHERAEGRHSTGGMDELYKPRKVYPIILRLALSYETEILTVEY-GLLPIGKIVEKRIECTVYSVDNNGNIYTQPVAQWHDRGEQEVFEYCLEDGSLIRAT-KDHKFMTVDGQMLPIDEIFERELDLMRVDNLPNIKIATRKYL-GKQNVVYDIGVERKRPAATKKAGQAKKKKLD

GR-Npu$_C$-mNES$_C$-2xFLAG (4)
Expected Mass (kDa): 92.72
Sequence:
MDSKESLTPGREENPSSVLAQERGDVMDFYKTLRGGAT-VKVSASSPSLAVASQSDSKQRRLLVDFPKGSVSNAQQPDLSKAVSLSMGLYMGE-TETKVMGNDLGFPQQGQISLSSGETDLKLLEESIANLNRSTSVPEN-PKSSASTAVSAAPTEKEFPKTHSDVSSEQQHLKGQTGTNGGNVKLYTTDQSTFD-ILQDLEFSSGSPGKETNESPWRSDLLIDENCLLSPLAGEDDSFLLEGNSNEDCK-PLILPDTKPKIKDNG-DLVLSSPSNVTLPQVKTEKEDFIELCTPGVIKQEKLGTVYCQASFPGANIIGNK-MSAISVHGVSTSGGQMYHYDMNTASLSQQQDQKPIFNVIPPIPVGSENWN-RCQGSGDDNLTSLGTLNFPGRTVFSNGY-SSPSMRPDVSSPPSSSSTATTGPPPKLCLVCSDEASGCHYGVLTCGSCKVFFKRAVE-GQHNYLCAGRNDCIIDKIRRKNCPACRYRKCLQAGMNLEARKTKKKIKGIQQATT-GVSQETSENPGNKTIVPATLPQLTPTLVSLLEV-IEPEVLYAGYDSSVPDSTWRIMTTLNMLGGRQVIAAVKWAKAIPG-FRNLHLDDQMTLLQYSWMFLMAFALGWRSYRQSSANLLCFAPDLI-INEQRMTLPCMYDQCKHMLYVSSELHRLQVSY-EEYLCMKTLLLLSSVPKDGLKSQELFDEIRMTYIKELGKAIV-KREGNSSQNWQRFYQLTKLLDSMHEVVENLLNYCFQTFLDKTMSIEFPEMLAEIIT-NQIPKYSNGNIKKLLFHQKLEIKIATRKYLGKQNVVYDIGVERDHNFALKNG-FIASNCFNLSLASDYKDDDDKDYKDDDDK

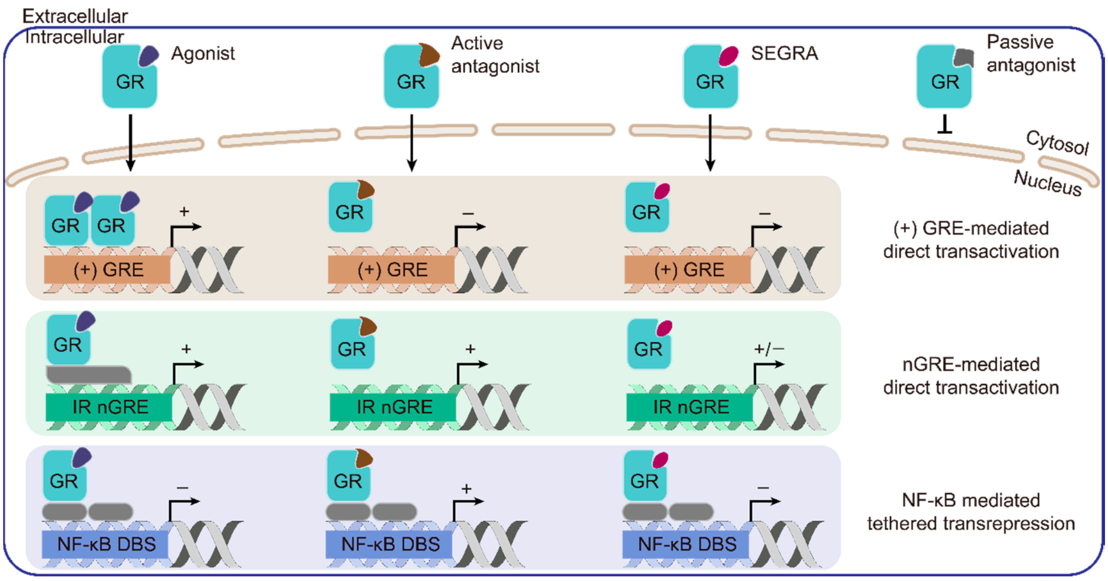

Scheme S1. A schematic illustration of the mode of GR gene regulation. Plus (+) indicates stimulation of gene expression and minus (-) in GRE or nGRE mediated GR transactivation suggests no effect on gene while the minus (-) in NF-kB mediated tethered transrepression implies repression of gene expression.

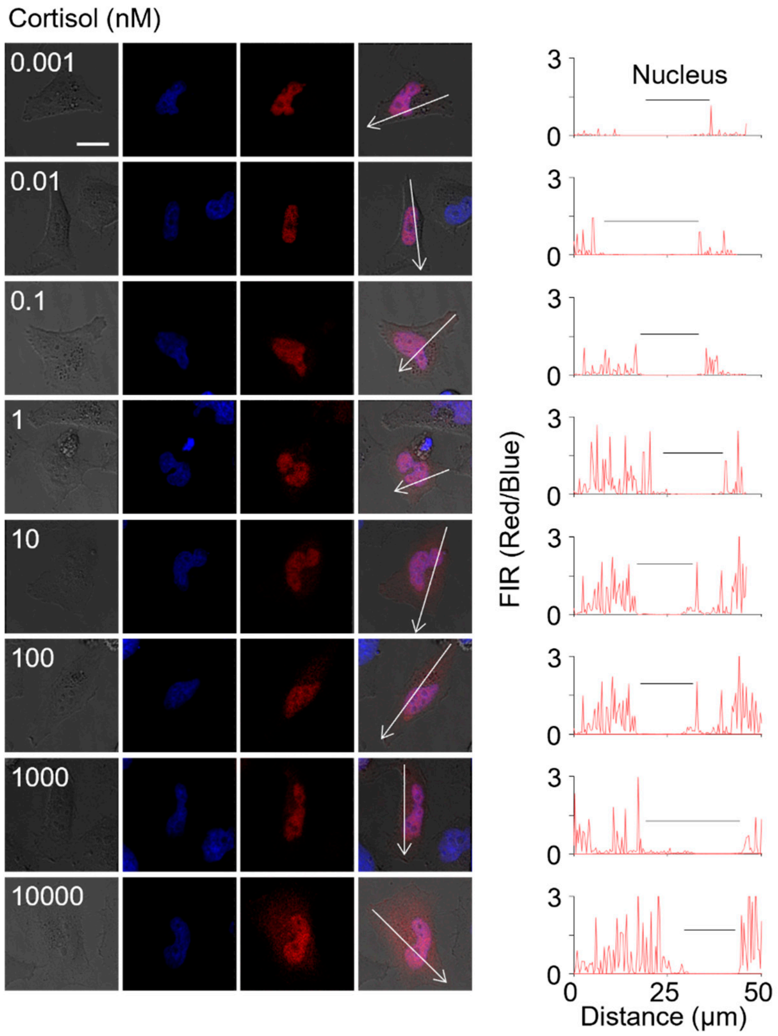

Figure S1. Dose-dependent treatment of cortisol to sensor cells. Cortisol was treated to the sensor cell by concentration and analyzed (scale bar = 20μm).

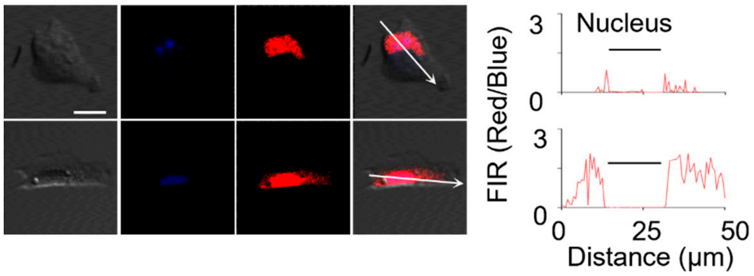

Figure S2. Detection of salivary cortisol using sensor cell. The sensor cells responded to Cort in human saliva with estimated Cort-concentration of 13 nM.

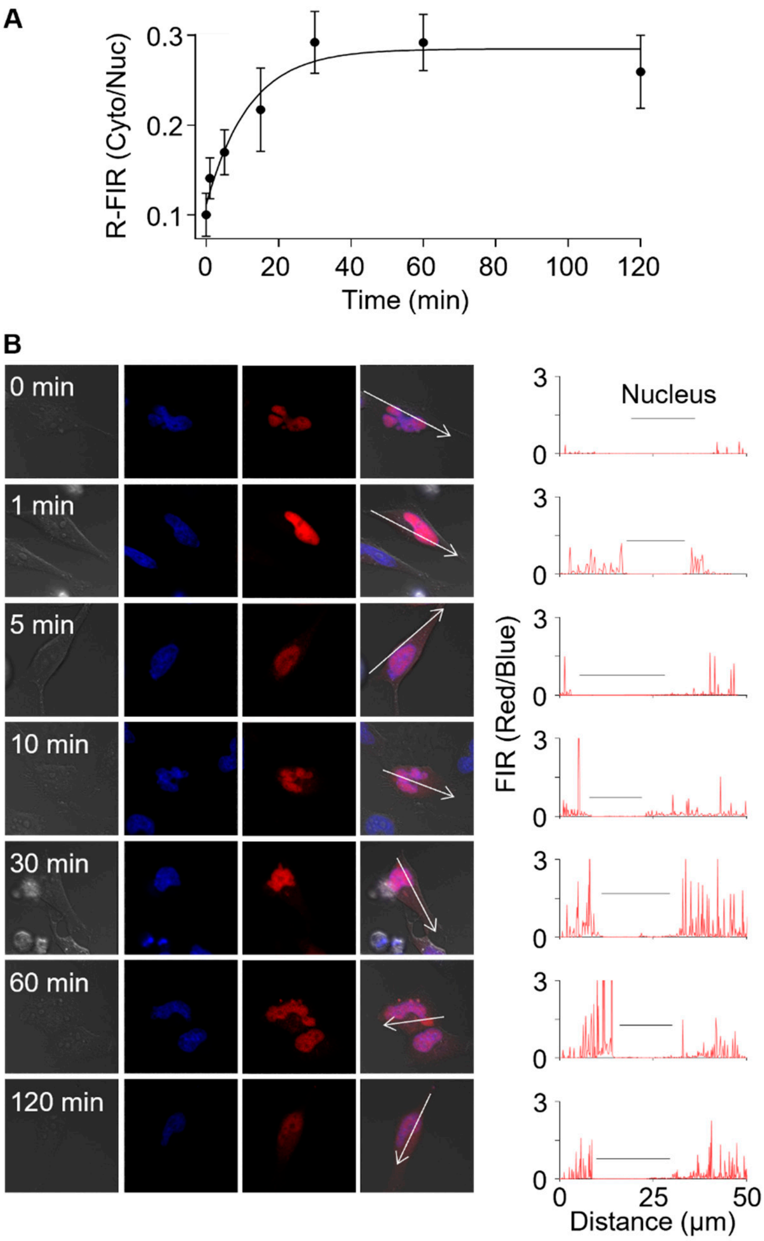

Figure S3. Time-dependent treatment of cortisol to sensor cells. (A) Time-dependent of R-FIR Cyto/Nuc signal was plotted and the limit of detection. (B) The sensor cells were treated with 10 μM of cortisol depending on time. (scale bar = 20μm)..

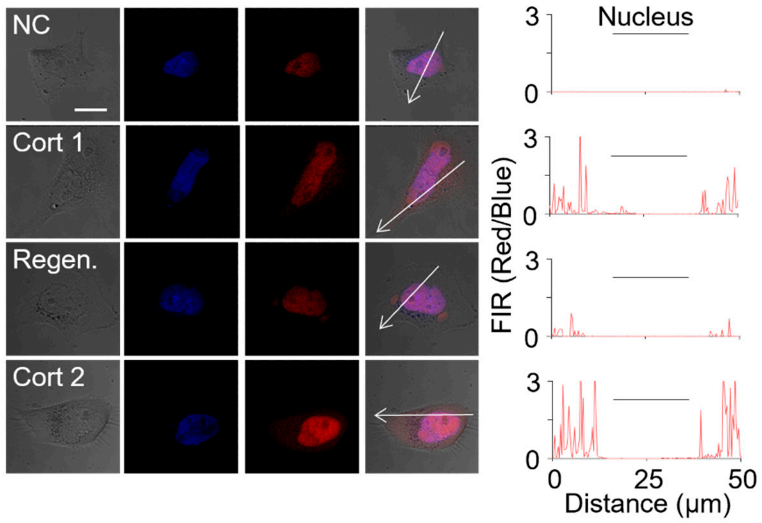

Figure S4. Renewability of sensor cell. Sensor cells were exposed to repetitive 10 μM of cortisol with time intervals (scale bar = 20μm).

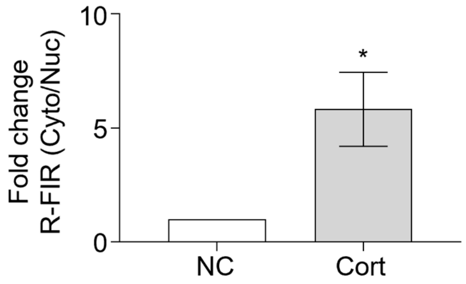

Figure S5. Cortisol sensing using HEK293T derived sensor cell. HEK293T-derived sensor cells successfully respond to cortisol and data were analyzed by unpaired T-test (* $p<0.05$).

Table S1. Table of medicinal plants and extraction conditions.

Common name	Biological name	ratio (w/v)[a]	time (h)
Gogi berry	Lycium chinense	1:10	2.5
Cornelian berry	Cornus officinalis	1:10	3
Black raspberry	Rubus coreanus	1:2	3
Tumeric	Curcuma longa	1:10	2
Dong-quai	Angelica gigas	1:6	3
Ginseng[b]	Panax ginseng	-	-
Hasuo	Polygonum muliflorum	1:12.5	3
Licorice	Glycyrrhiza uralensis	1:10	3
Deer antler[b]	Cervus	-	-
Red ginseng	Panax ginseng	1:10	24
Reishi mushroom	Ganoderma lucidum	1:20	24
Red bean	Vigna angularis	1:20	24
Onion	Alluium cepa	1:10	12
Garlic	Allium sativum	1:10	3

[a] Ratio of the weight of the natural product and the volume of 70 % ethanol

[b] Purchased from Dongguk University Medical Center (Goyang, Korea)
